
JOURNAL INFORMATION
==============================
NLM Title Abbreviation: Biospektrum (Heidelb)
Journal ID: Biospektrum (Heidelb)
NLM Journal ID: 9615685

Biospektrum

ISSN: 0947-0867
EISSN: 1868-6249

ARTICLE INFORMATION
==============================
PMCID: PMC7686442
PMID: 33250577
DOI: 10.1007/s12268-020-1491-2
Article ID: 1491
Article version: 1
Subjects: Wissenschaft Special

Protein-AMPylierungs-Identifikation in lebenden Zellen

Becker Tobias
Kielkowski Pavel pavel.kielkowski@cup.lmu.de http://www.cup.lmu.de/oc/kielkowski

grid.5252.0 0000 0004 1936 973X Department Chemie, Ludwig-Maximilians-Universität München, Butenandtstraße 5-13, D-81377 München, Deutschland
Electronic publication date: 2020 Nov 25
Print publication date: 2020
Volume: 26
Issue: 7
First page: 743
Last page: 746
Copyright: © Die Autoren 2020
License: Dieser Artikel wird unter der Creative Commons Namensnennung 4.0 International Lizenzveröffentlicht, welche die Nutzung, Vervielfältigung, Bearbeitung, Verbreitung und Wiedergabe in jeglichem Medium und Format erlaubt, sofern Sie den/die ursprünglichen Autor(en) und die Quelle ordnungsgemäß nennen, einen Link zur Creative Commons Lizenz beifügen und angeben, ob Änderungen vorgenommen wurden. Die in diesem Artikel enthaltenen Bilder und sonstiges Drittmaterial unterliegen ebenfalls der genannten Creative Commons Lizenz, sofern sich aus der Abbildungslegende nichts anderes ergibt. Sofern das betreffende Material nicht unter der genannten Creative Commons Lizenz steht und die betreffende Handlung nicht nach gesetzlichen Vorschriften erlaubt ist, ist für die oben aufgeführten Weiterverwendungen des Materials die Einwilligung des jeweiligen Rechteinhabers einzuholen. Weitere Details zur Lizenz entnehmen Sie bitte der Lizenzinformation auf http://creativecommons.org/licenses/by/4.0/deed.de.
License URL: https://creativecommons.org/licenses/by/4.0/

issue-copyright-statement: © Springer-Verlag GmbH Deutschland, ein Teil von Springer Nature 2020

==============================
Protein AMPylation is a prevalent protein post-translational modification in human cells involved in endoplasmic reticulum stress regulation and neural development. In this article we describe the design, synthesis and application of a pronucleotide probe suitable for in situ fluorescence imaging and chemical protemics profiling of AMPylated proteins. Our probe utilizes straightforward strain-promoted azidealkyne click reaction for fluorescence labeling in living cells.

■ Das menschliche Proteom ist um ein Vielfaches komplexer als das menschliche Genom. Ein Grund hierfür ist, dass Proteine mit bestimmten funktionellen Gruppen modifiziert werden, um ihre Aktivität, Lokalisierung und Interaktion mit anderen Proteinen zu regulieren [1]. Es gibt unterschiedlichste dieser posttranslationalen Modifikationen und eine Vielzahl von ihnen ist bereits gut erforscht. In diesem Artikel fokussieren wir uns auf eine posttranslationale Modifikation, der in jüngster Zeit immer größere Aufmerksamkeit zuteil wird: die AMPylierung [2, 3]. Charakteristisch für diese ist die Übertragung eines Adenosinmonophosphats (AMP) auf eine alkoholische Aminosäureseitenkette, wie Serin, Threonin oder Tyrosin. Diese Reaktion wird von AMP-Transferasen (AMPylatoren) katalysiert, die Adenosintriphosphat (ATP) als Ko-Substrat verwenden (**Abb. 1A**).

Zur genaueren Untersuchung der AMPylierung wurden bereits unterschiedliche Methoden eingesetzt, wie etwa strukturell veränderte ATP-Analoga in Zelllysaten. Dies hat allerdings einen großen Nachteil: Die ATP-Analoga weisen aufgrund von Phosphatresten negative Ladungen auf, wodurch sie die Zellmembran nicht passieren können. Die Analyse der AMPylierung mit ATP-Analoga in lebenden Zellen ist aus diesem Grund nicht möglich. Um diese Problematik zu umgehen, entwickelte unsere Forschungsgruppe eine zellpermeable Sonde (pro-N6pA), die anstelle eines Triphosphats eine Phosphoramidatgruppe enthält (**Abb. 1B**, [4]). Die Zellpermeabilität basiert darauf, dass die Phosphoramidatgruppe im Gegensatz zur Triphosphatgruppe keine negative Ladung aufweist. Sobald die pro-N6pA-Sonde die Zellmembran passiert, wird sie metabolisch aktiviert, wodurch das aktive N6pATP entsteht. In der medizinischen Chemie ist diese Methode bereits weit verbreitet, etwa um antivirale Medikamente in die Zelle zu transportieren, wie Sofosbuvir gegen Hepatitis C oder Remdesivir, dessen Wirksamkeit gegen das neuartige Coronavirus derzeit getestet wird [5].

Eine weitere Besonderheit der aktivierten N6pATP-Sonde ist ihre Alkingruppe, die sowohl eine Kopplung mit einem Affinitätstag als auch einem Fluoreszenzfarbstoff zulässt. Hierfür wird die „Click-Chemie“ verwendet, die schnell und bioorthogonal ist. Für diese Reaktion wird jedoch Kupfer(I) benötigt, das zytotoxisch ist und damit verhindert, dass AMPylierte Proteine auch in lebenden Zellen fluoreszenzmarkiert werden können. Um die Fluoreszenzmarkierung AMPylierter Proteine dennoch zu ermöglichen, entwickelte unsere Forschungsgruppe eine weitere Sonde (pro-N6azA), die anstelle eines Alkins ein Azid enthält (**Abb. 1C**, [6]). Somit kann statt der bisher verwendeten kupferbasierten und zytotoxischen Click-Chemie entweder die kupferfreie *strain-promoted azide-alkyne cycloaddition* (SPAAC) oder die Staudinger-Ligation verwendet werden [7].

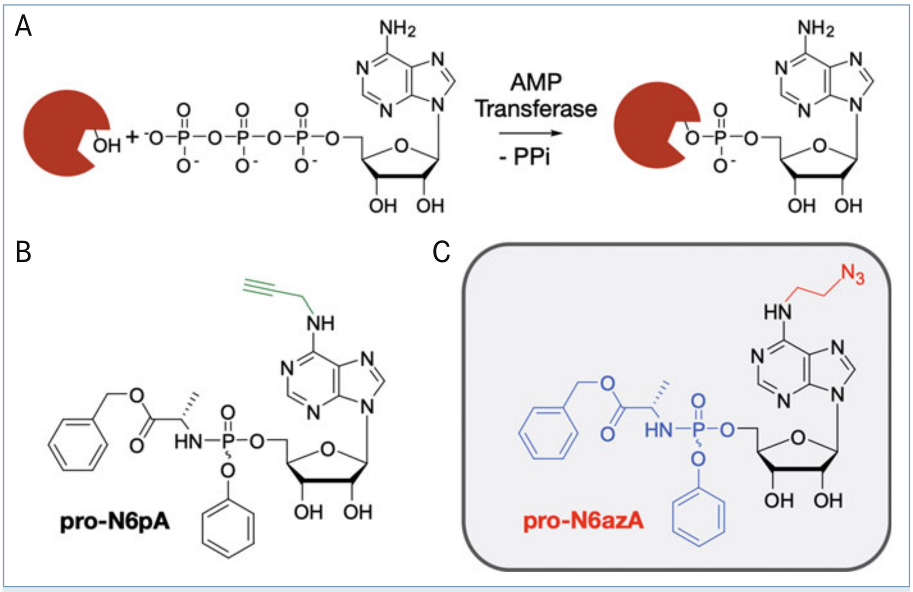

▲ **Abb. 1:** Protein-AMPylierung und Sonden, die zur Markierung in lebenden Zellen geeignet sind. **A**, schematische Darstellung der Protein-AMPylierung. **B**, zuvor veröffentlichte Sonde pro-N6pA. **C**, Struktur der in dieser Studie vorgestellten Sonde pro-N6azA.

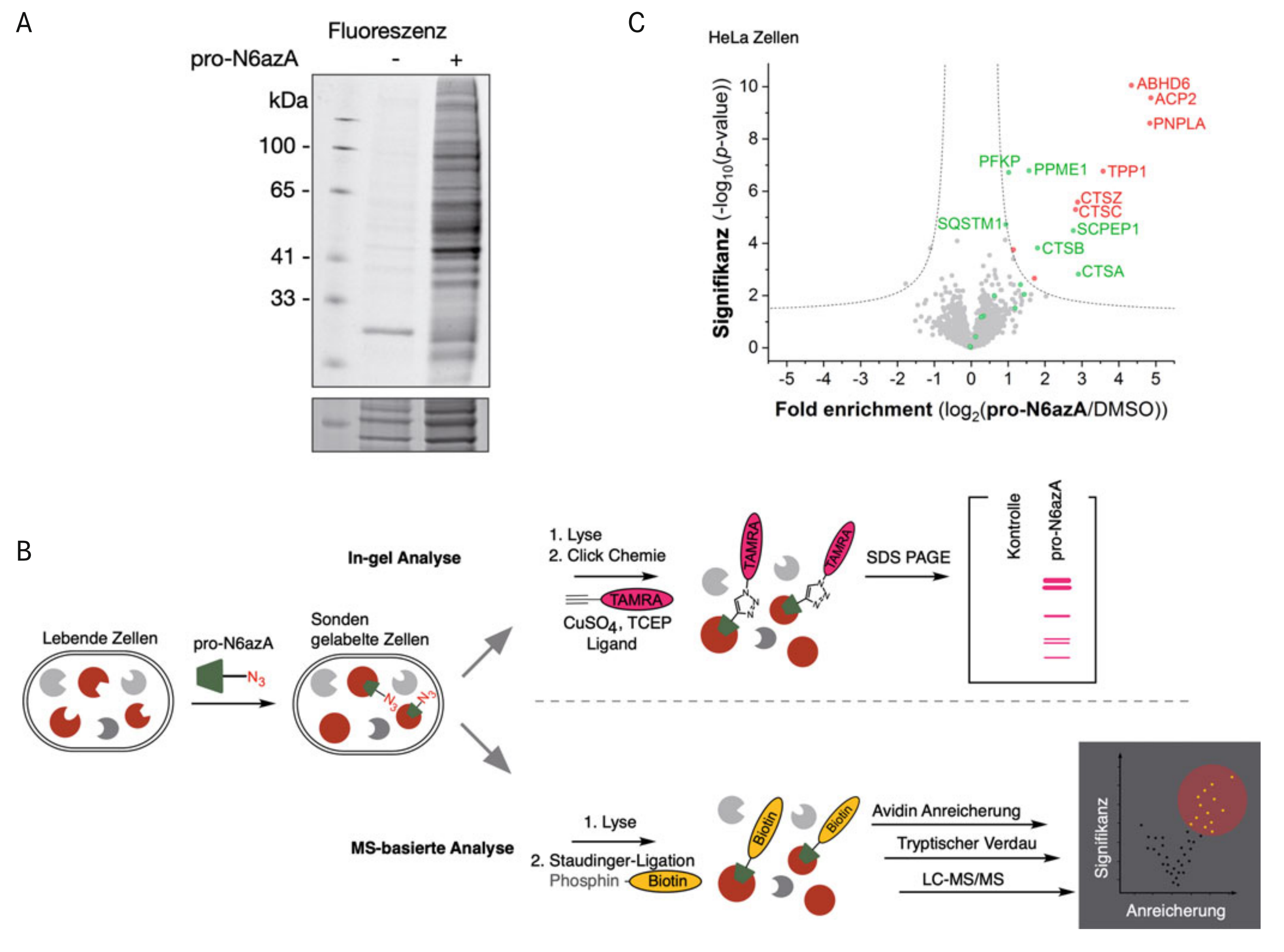

▲ **Abb. 2:** Identifizierung von AMPylierungs-Zielen durch chemische Proteomik-Experimente. **A**, Fluoreszenzscan von SDS-PAGE-getrennten Proteinen, die mit der pro-N6azA-Sonde in lebenden Zellen behandelt wurden und mit einem Fluoreszenzfarbstoff markiert wurden. **B**, schematische Darstellung der chemischen Proteomik-Methode. HeLa-Zellen wurden zunächst über einen Zeitraum von 16 Stunden mit 100 µM der pro-N6azA-Sonde behandelt. Im Anschluss wurden die modifizierten Proteine entweder an einen Biotin-Affinitätstag oder einen Fluoreszenzfarbstoff gekoppelt. Der Fluoreszenzfarbstoff wurde dabei zur Visualisierung der AMPylierten Proteine verwendet, wobei der Biotin-Affinitätstag zur Anreicherung und anschließenden Analyse mittels Massenspektrometrie diente. **C**, *volcano plot* mit pro-N6azA-angereicherten Proteinen. Proteine, die zuvor mit der pro-N6pA-Sonde identifiziert wurden, sind grün hervorgehoben. Proteine, die zusätzlich mit der pro-N6azA-Sonde identifiziert wurden, sind rot hervorgehoben.

Da die neu synthetisierte Sonde pro-N6azA in lebenden Zellen verwendet wird, musste zunächst Ihre Zytotoxizität mittels MTT-Test bestimmt werden. In HeLa-Zellen (humane Krebszelllinie) wurde hierbei ein $IC_{50}$ von 239,2 Mikromol gemessen, was bedeutet, dass bei dieser Konzentration der Sonde 50 Prozent der Zellen sterben. Deswegen entschieden wir uns dafür, die darauffolgenden Experimente mit einer Konzentration von 100 Mikromol durchzuführen, um eine möglichst hohe Sondenkonzentration bei ausbleibender Zytotoxizität sicherzustellen. Eine hohe Sondenkonzentration ist notwendig, damit N6azATP mit dem natürlich in der Zelle vorkommenden ATP konkurrieren kann.

Nachdem die geeignete Konzentration der pro-N6azA-Sonde festgelegt war, konnte im nächsten Schritt untersucht werden, ob die Sonde von endogenen AMPylatoren akzeptiert wird. Der hierzu verwendete Ansatz basiert darauf, dass AMPylierte Zielproteine bei erfolgreicher Aktivierung der Sonde einen zusätzlichen Azidrest aufweisen, der mittels SPAAC an einen Fluoreszenzfarbstoff gekoppelt werden kann. In der Folge kann dieser mittels Polyacrylamid-Gelelektrophorese (SDS-PAGE) und einer geeigneten Kamera mit Fluoreszenzfilter detektiert werden. Die SDS-PAGE-Analyse zeigt, dass die pro-N6azA-Sonde erfolgreich von endogenen AMPylatoren als Substrat verwendet wurde, da ohne Einsatz der Sonde keine Fluoreszenz-Proteinbanden zu beobachten waren (**Abb. 2A**).

Damit war die Voraussetzung für die anschließenden chemischen Proteomik-Experimente geschaffen [8], durch die die Zielproteine der pro-N6azA-Sonde genauer identifiziert werden sollten (**Abb. 2B**). Hierfür wurden intakte HeLa-Zellen über einen Zeitraum von 16 Stunden mit einer Konzentration von 100 Mikromol der pro-N6azA-Sonde behandelt, was – wie oben beschrieben – bewirkt, dass die AMPylierten Zielproteine einen zusätzlichen Azidrest tragen. Im Anschluss wurden die modifizierten Proteine mittels Staudinger-Ligation an einen Biotin-Affinitätstag gekoppelt, der eine darauffolgende Affinitätsanreicherung an Avidin-

Agarose *beads* erlaubt. Die so angereicherten Proteine wurden durch das Verdauungsenzym Trypsin zu kleineren Peptidfragmenten abgebaut und konnten schließlich mittels einer Kombination aus einem Massenspektrometer und Flüssigkeitschromatographie analysiert werden.

Zur Erfassung der AMPylierungs-Ziele wurde die *label-free quantification* (LFQ) eingesetzt [9], die auch immer Kontrollexperimente für einen Vergleich der veränderten AMPylierungs-Niveaus benötigt. Hierzu wurden HeLa-Zellen mit Dimethylsulfoxid (DMSO), dem Lösemittel der pro-N6azA-Sonde, behandelt, um auszuschließen, dass unspezifisch angereicherte Proteine fälschlicherweise als AMPylierungs-Ziele kategorisiert werden. Die Ergebnisse zur Identifizierung der Zielproteine der pro-N6azA-Sonde sind in einem *volcano plot* dargestellt, der sich dadurch auszeichnet, dass er sowohl die Signifikanz als auch die Stärke der Anreicherung visualisiert. Das heißt, je signifikanter und stärker ein Protein angereichert wurde, desto weiter rechts und oben ist dieses im *volcano plot* zu sehen. In unseren Experimenten wurde deutlich, dass sowohl bereits bekannte (grün markiert) als auch bisher unidentifizierte (rot markiert) AMPylierungs-Ziele bei Verwendung der pro-N6azA-Sonde signifikant angereichert wurden (**Abb. 2C**). Somit konnten wir belegen, dass sich die pro-N6azA-Sonde zur Identifizierung von AMPylierungs-Zielen in lebenden Zellen eignet. Damit wurde die Grundlage zur Visualisierung der AMPylierung mittels Fluoreszenzmikroskopie in lebenden Zellen geschaffen, welche bisher noch nicht möglich war. Die Visualisierung würde es ermöglichen, die Dynamik der AMPylierung in lebenden Zellen zu überwachen und so dazu beitragen, die Funktion dieser posttranslationalen Modifikation in zellulären Schlüsselprozessen aufzuklären.

Zur Etablierung der neuen Methode wurde die Detektion der Fluoreszenzmarkierung mittels Fluoreszenzmikroskopie zunächst durch Kontrollexperimente in fixierten Zellen überprüft. Hierzu wurden im ersten Schritt HeLa-Zellen über einen Zeitraum von 16 Stunden mit der pro-N6azA-Sonde behandelt und anschließend fixiert. Im Anschluss wurden die fixierten Zellen mithilfe von Click-Chemie an einen Fluoreszenzfarbstoff gekoppelt und unter einem Fluoreszenzmikroskop visualisiert. Dabei zeigte sich einerseits, dass die Fluoreszenzmarkierung mittels Fluoreszenzmikroskopie gut sichtbar ist, und andererseits, dass der Großteil der Modifikation im Zellkern lokalisiert ist (**Abb. 3A**). Dieser Rückschluss ist zulässig, da die Fluoreszenzmarkierung mit dem Fluoreszenzfarbstoff DAPI kolokalisiert, der sich an DNA anlagert (**Abb. 3A**). Ein kleiner Teil der AMPylierten Proteine findet sich auch im Cytoplasma, was wiederum gut mit vorherigen Studien an der pro-N6pA-Sonde übereinstimmt.

Nachdem wir in den Kontrollexperimenten erfolgreich demonstrieren konnten, dass die Fluoreszenzmarkierung durch pro-N6azA in fixierten Zellen funktioniert, sollte im nächsten Schritt untersucht werden, ob dies auch in lebenden Zellen möglich ist. Dazu wurden sowohl HeLa-Zellen als auch Fibroblasten 16 Stunden lang mit der pro-N6azA-Sonde behandelt und im Anschluss ohne Fixierung direkt in lebenden Zellen mittels SPAAC an einen Fluoreszenzfarbstoff gekoppelt. Hierbei ist nochmals hervorzuheben, dass SPAAC im Gegensatz zur klassischen Click-Chemie kein toxisches Kupfer(I) zur Katalyse benötigt und damit in lebenden Zellen verwendet werden kann. Nach der Kupplungsreaktion wurden die Zellen für weitere 24 Stunden inkubiert und anschließend mithilfe von Fluoreszenzmikroskopie analysiert. Dabei wurde erkennbar, dass beide Zelllinien erfolgreich fluoreszenzmarkiert wurden. Dies beweist auf der einen Seite, dass die pro-N6azA-Sonde in der Lage ist, die AMPylierung in lebenden Zellen zu visualisieren und auf der anderen Seite, dass die AMPylierung eine stabile posttranslationale Modifikation ist (**Abb. 3B**). Zudem fällt auf, dass die AMPylierten Proteine in Fibroblasten im Gegensatz zu HeLa-Zellen hauptsächlich im Cytoplasma vorliegen.

Damit war bewiesen, dass sich die pro-N6azA-Sonde zur Fluoreszenzmarkierung AMPylierter Proteine in lebenden Zellen eignet und wir konnten uns im Folgenden auf das finale Ziel unsere Studie konzentrieren – nämlich die Dynamik der AMPylierung in angemessener räumlicher und zeitlicher Auflösung zu visualisieren. Hierzu wurden Fibroblasten mit der pro-N6azA-Sonde behandelt und über einen Zeitraum von 25 Minuten alle 20 Sekunden Momentaufnahmen mit einem konfo-

Hier steht eine Anzeige.
Springer

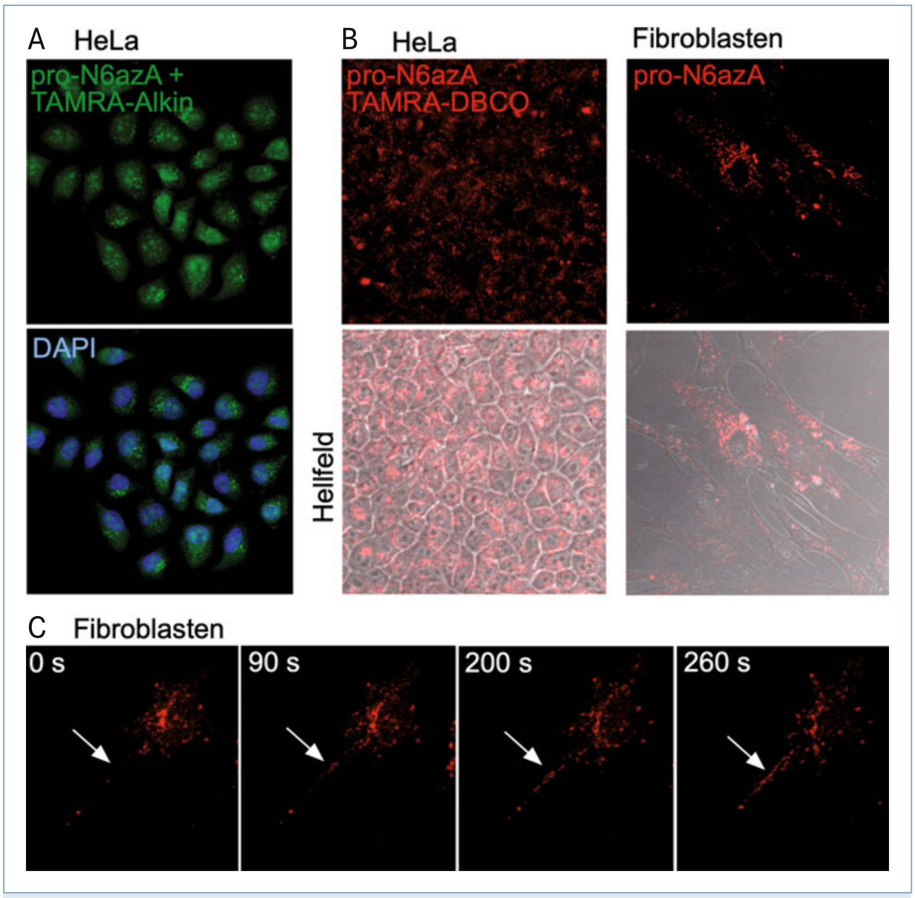

▲ **Abb. 3:** Visualisierung AMPylierter Proteine mittels Fluoreszenzmarkierung in fixierten und lebenden Zellen. **A**, AMPylierungs-Ziele in Paraformaldehyd-fixierten HeLa-Zellen wurden mit der pro-N6azA-Sonde und einem Alkin-Fluoreszenzfarbstoff (TAMRA-Alkin) markiert. AMPylierte Proteine sind grün dargestellt, der Zellkern blau. **B**, pro-N6azA-behandelte lebende HeLa- und Fibroblastenzellen, welche mittels SPAAC an einen Fluoreszenzfarbstoff (TAMRA-DBCO) gekoppelt wurden. Hierbei sind die AMPylierten Proteine rot dargestellt. **C**, Zeitrafferaufnahmen des gerichteten Transports AMPylierter Proteine zu den Enden der Zellkörper von Fibroblasten. Die AMPylierten Proteine sind rot dargestellt, zugleich weisen weiße Pfeile auf den Transport innerhalb der Zelle hin.

kalen Laser-Scanning-Mikroskop aufgenommen (**Abb. 3C**). Dabei zeigte sich, dass sich die Lokalisierung der AMPylierten Proteine über die Zeit veränderte und diese gerichtet vom Zentrum der Zelle an die Enden der Zellkörper transportiert wurden. Diese Beobachtung lässt zum einen darauf schließen, dass die AMPylierung möglicherweise eine Rolle bei der Zellpolarisierung spielt und zum anderen, dass die pro-N6azA-Sonde geeignet ist, die Dynamik der AMPylierung in lebenden Zellen nachzuverfolgen. Damit eröffnen sich für die Zukunft viele neue Möglichkeiten zur besseren Charakterisierung dieser weitgehend uncharakterisierten posttranslationalen Modifikation.

Zusammenfassend ist festzuhalten, dass es uns gelungen ist, mit der pro-N6azA-Sonde ein leistungsfähiges chemisch-biologisches Werkzeug für zukünftige Studien zur Untersuchung der Protein-AMPylierung zu etablieren. Hierbei ist insbesondere hervorzuheben, dass die Sonde es ermöglicht, die Dynamik der Protein-AMPylierung in lebenden Zellen unter einem konfokalen Laser-Scanning-Mikroskop zu verfolgen. ■


Funding note

Open Access funding enabled and organized by Projekt DEAL.

Tobias Becker 2013–2017 Bachelorstudium Chemie und Biochemie an der LMU München. 2017–2019 Masterstudium Biochemie an der TU München. Seit 2019 Promotion an der LMU München in der Nachwuchsgruppe von Dr. Kielkowski.

Pavel Kielkowski 2007–2009 Masterstudium Chemie der Naturstoffe am Institute of Chemical Technology, Prag, Tschechien. 2009–2014 Promotion Organische Chemie am Institute of Organic Chemistry and Biochemistry AS CR (IOCB), Prag und an der Charles University in Prag. 2014–2019 Postdoktorand an der TU München in der Gruppe von Prof. Dr. S. A. Sieber. Seit 2019 Nachwuchsgruppenleiter an der LMU München.


Literatur

[1] Aebersold R Agar JN Amster IJ How many human proteoforms are there? Nat Chem Biol 2018 14 206 214 10.1038/nchembio.2576 29443976 PMC5837046
[2] Casey AK Orth K Enzymes involved in AMPylation and deAMPylation Chem Rev 2017 118 1199 1215 10.1021/acs.chemrev.7b00145 28819965 PMC5896785
[3] Sieber SA Cappello S Kielkowski P From young to old: AMPylation hits the brain Cell Chem Biol 2020 27 773 779 10.1016/j.chembiol.2020.05.009 32521229
[4] Kielkowski P Buchsbaum IY Kirsch VC FICD activity and AMPylation remodelling modulate human neurogenesis Nat Commun 2020 11 517 10.1038/s41467-019-14235-6 31980631 PMC6981130
[5] Mehellou Y Rattan HS Balzarini J The ProTide prodrug technology: from the concept to the clinic J Med Chem 2018 61 2211 2226 10.1021/acs.jmedchem.7b00734 28792763 PMC7075648
[6] Kielkowski P Buchsbaum IY Becker T A pronucleotide probe for live cell imaging of protein AMPylation Chembiochem 2020 21 1285 1287 10.1002/cbic.201900716 32027064 PMC7317759
[7] Agard NJ Prescher JA Bertozzi CR A strain-promoted [3 + 2] azide alkyne cycloaddition for covalent modification of biomolecules in living systems J Am Chem Soc 2004 126 15046 15047 10.1021/ja044996f 15547999
[8] Speers AE Adam GC Cravatt BF Activity-based protein profiling in vivo using a copper(I)-catalyzed azidealkyne [3 + 2] cycloaddition J Am Chem Soc 2003 125 4686 4687 10.1021/ja034490h 12696868
[9] Cox J Hein MY Luber CA Accurate proteome-wide label-free quantification by delayed normalization and maximal peptide ratio extraction, termed MaxLFQ Mol Cell Proteomics 2014 13 2513 2526 10.1074/mcp.M113.031591 24942700 PMC4159666
